# ASSOCIATIONS OF ADVERSE CHILDHOOD EXPERIENCES WITH DSM-5 DEPRESSIVE DISORDERS AND SUICIDE ATTEMPT IN OLDER ADULTS

**DOI:** 10.1093/geroni/igz038.2117

**Published:** 2019-11-08

**Authors:** Taeho Greg Rhee, Lisa C Barry, George A Kuchel, David C Steffens, Samuel Wilkinson

**Affiliations:** 1 University of Connecticut School of Medicine, Farmington, Connecticut, United States; 2 Yale University School of Medicine, New Haven, Connecticut, United States

## Abstract

Adverse childhood experiences (ACEs) may have long-term effects on mental health. Using a life-course perspective, this study examines prevalence of ACEs and the associations of ACEs with depressive disorders and suicide attempt in US older adults. The study sample were those aged 65 and older who participated in the 2012-2013 National Epidemiological Survey on Alcohol and Related Conditions Wave III (n=5,806 unweighted). ACEs, the key independent variable, were assessed using validated measures and outcome variables included lifetime and past-year major depressive disorder (MDD) and dysthymia using DSM-5 criteria, and lifetime suicide attempt. We estimated national prevalence of ACEs in older adults and used multivariable-adjusted logistic regression analyses to assess the association between ACEs and the outcomes after adjusting for socio-demographics and clinical co-morbidities. Overall, 34.7% of older adults, representative of 14.3 million older adults nationwide, reported some form of ACEs. The most common type was parental psychopathology (20.8%), followed by neglect (14.8%), and physical/psychological abuse (8.4%) (non-mutually exclusive). Having experienced any ACEs was associated with higher odds of having a past-year MDD diagnosis (adjusted odds ratio [aOR]=1.77; 95% confidence intervals [CI]=1.36, 2.29). Similar results were found for other depressive disorders. ACEs were also associated with higher odds of having a lifetime suicide attempt (aOR=4.34; 95% CI=2.64, 7.14). In conclusion, ACEs may expose older adults to an increased risk for mood disorders and suicide attempts, even over long periods of time as seen in this sample. Reducing ACEs is an important public health goal that may yield long-term benefits.

